# Location of Sternal Fractures as a Possible Marker for Associated Injuries

**DOI:** 10.1155/2013/407589

**Published:** 2013-11-13

**Authors:** Max J. Scheyerer, Stefan M. Zimmermann, Samy Bouaicha, Hans-Peter Simmen, Guido A. Wanner, Clément M. L. Werner

**Affiliations:** Division of Trauma Surgery, Department of Surgery, University Hospital Zurich, Raemistraße 100, 8091 Zürich, Switzerland

## Abstract

*Introduction*. Sternal fractures often occur together with serious and life-threatening additional injuries. This retrospective study was designed to assess concomitant injuries and develop a correlation between fracture location and the severity of injury. *Methods.* All patients (*n* = 58) diagnosed with a fracture of the sternum by means of a CT scan were analysed with respect to accident circumstances, fracture morphology and topography, associated injuries, and outcome. *Results*. Isolated sternal fractures occurred in 9%. In all other admissions, concomitant injuries were diagnosed: mainly rip fractures (64%), injury to the head (48%), the thoracic spine (38%), lumbar spine (27%), and cervical spine (22%). Predominant fracture location was the manubrium sterni. In these locations, the observed mean ISS was the highest. They were strongly associated with thoracic spine and other chest injuries. Furthermore, the incidence of head injuries was significantly higher. ICU admission was significantly higher in patients with manubrium sterni fractures. *Conclusion*. Sternal fractures are frequently associated with other injuries. It appears that the fracture location can provide important information regarding concomitant injuries. In particular, in fractures of manubrium sterni, the need for further detailed clinical and radiologic workup is necessary to detect the frequently associated injuries and reduce the increased mortality.

## 1. Introduction

The most common mechanisms accounting for sternal fractures are motor vehicle collisions and blunt trauma to the chest and abdomen [1–3]. During the last decades, the detection rate of this injury has increased due to the obligation to wear a seat belt in cars as well as improved imaging through the common use of computed tomography in the emergency room after accidents. Today, a fracture of the sternum is observed in 4% of all traffic accident victims and 3–8% following blunt abdominal trauma [4]. 

In the past, the general belief was that a sternum fracture represented a serious injury due to commonly associated potentially life-threatening injuries. Some authors have reported mortality rates in patients with sternal fractures ranging from 24% up to as much as 45% [5, 6]. This high mortality rate is due to associated thoracic, pulmonary, cardiac, and spinal injuries [7–11]. Other studies, however, have shown that only one-third of all patients with sternal fractures in fact also suffered from concomitant injuries [12]. The remaining patients sustained isolated sternal fractures which can be classified as harmless injuries. Treatment options for this minor injury are therefore analogous to isolated rib fractures, consisting of conservative therapy in an ambulatory setting. 

With regard to the further treatment, it is therefore important to distinguish between isolated harmless and associated serious sternal fractures. 

Several studies in the past have investigated concomitant injuries in patients which had sustained a sternal fracture [13–15]. However, these studies were not able to demonstrate a significant correlation between sternum fracture morphology and associated injuries [8, 16, 17]. 

The aim of our current study was to assess whether by means of a simple subdivision of the sternum a correlation between the location of a sternal fracture and specific concomitant injuries could be demonstrated. In this case, the location of a sternal fracture could serve as a possible indicator for serious additional injuries. We therefore retrospectively reviewed a series of patients with an injury of the sternum over the course of a four-year period and analysed the fracture location, associated spinal fractures, and other concomitant injuries.

## 2. Patients and Methods

Patients with a fracture of the sternum who were initially admitted to the emergency ward of a trauma 1 center for assessment between March 2007 and June 2011 were included in this study. 

Further inclusion criteria were presence of a whole body computed tomography performed with contrast (SOMATOM Definition, Siemens, Munich, Germany; 128-slice dual source CT; 120 kV, 210 mAs, slice thickness 3 mm).

The diagnosis of a sternal fracture was confirmed when a cortical disruption with or without displacement was detected. The analysis of the CT scans was performed by an orthopaedic surgeon and in borderline cases the senior author made a final decision.

**Figure 1:**
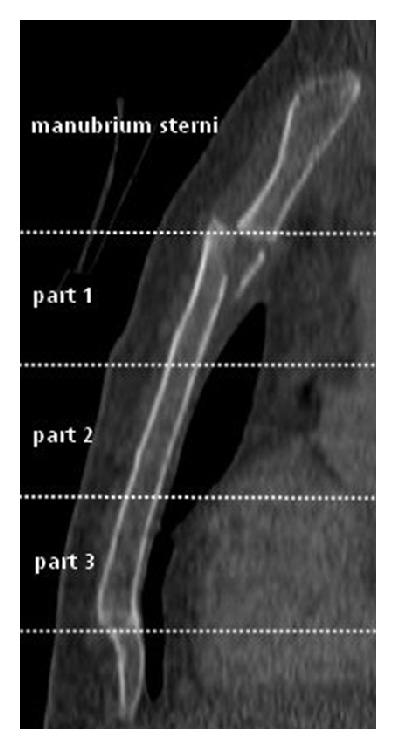
Topographic division of the sternum in four parts: the manubrium sterni and corpus sterni including parts 1, 2, and 3.

For evaluation of concomitant injuries the sternum was divided into four zones. Although, the topographic division is only artificial, the use is well described in previous studies [13, 18] (see Figure 1): the manubrium sterni, the upper part of corpus sterni (part 1), middle part of corpus sterni (part 2), and finally the distal corpus sterni including the xiphoid (part 3).

The following parameters were examined retrospectively: gender, age at time of injury, monotrauma or multiple injury, the injury severity score (ISS), and circumstances regarding the mechanism of injury. Furthermore, we analysed the ICU admission and mortality rate. 

Following this, concomitant injuries of the head, chest, spine, and abdomen were examined: head injuries were subdivided into three groups: first minor injuries to the head including cuts, secondly concussions, and finally intracranial hemorrhaging. Injuries of the chest included rib fractures and serial rib fractures, fractures of the clavicle and scapula as well as pneumothorace, lung contusions, and parenchymal lesions which were identified as focal areas of parenchymal opacification in the CT scan. Cardiac contusion was defined as a detection of elevated levels of CK-MB and troponin T or arrhythmia. In case of a spine fracture, the fracture was classified using the AO classification [19]. 

Abdominal injuries included hemorrhagic lesions of the spleen, liver, ovarian, kidneys, adrenal glands as identified on CT scans as well as lacerations of the stomach and smaller intestines, and finally lesions of the abdominal aorta. 

These injuries were each analysed according to the level of the accompanying sternum fracture. 

Post hoc tests were performed to evaluate differences in injury severity according to location of sternum fracture. Differences between fracture location and concomitant injuries were analyzed using chi-square tests and confidence intervals. A probability value of <0.05 was considered statistically significant. Analysis was performed using SPSS1 software (Version 18.0; SPSS Inc., Chicago, IL). Due to the retrospective nature of the study and the current local regulations no further approval of the local ethics committee was necessary.

## 3. Results

Between March 2007 and June 2011, a total of fifty-eight patients with sternal fractures were admitted to our department, of which thirty-two were men and twenty-six were women. The average age was 53 (range 18–94). Isolated sternal fractures were detected in 9% (*n* = 5) of the patients. In all other admissions, concomitant injuries were diagnosed (Table 2). Overall, the mean ISS was 20.5.

The most common mechanism of injury was motor vehicle collisions accounting for 43% (*n* = 25) of cases. The remaining causes are listed in Table 1. 

The predominant fracture location was in the manubrium sterni (*n* = 21) and the middle part of the corpus sterni (*n* = 15) (Table 3). Fractures of the upper as well as lower part of the corpus sterni were rare (*n* = 11). In three cases involvement of the synchondrosis manubriosternalis was diagnosed. The mean ISS was the highest in patients with a fracture of the manubrium (ISS = 23) or part 2 of the sternum (ISS = 22) compared to the remaining levels. However, these differences were not statistically significant (*P* = 0.744).

Overall, thirty-three patients (57%) with a fracture of the sternum were admitted to the intensive care unit. In case of fracture of the manubrium, the rate was significantly higher in contrast to other locations (*P* = 0.024) (Table 4). The overall mortality rate for all patients with a sternal fracture was 15.5% (*n* = 9). The highest rates could also be observed in patients with a fracture of the manubrium sterni and part 2 of the corpus sterni (Table 4). However the differences were not significant.

Concomitant injuries were found in 91% of the cases (*n* = 53). A head injury was found in almost half of the patients with a sternal fracture (*n* = 28/58, 48%). Of these, one patient presented with a minor injury, 15 suffered from concussions, and 12 cases of intracranial hemorrhaging were found. A head injury was most frequently associated with fractures of the manubrium sterni (*n* = 14/28). The difference to the remaining topographic zones was statistically significant especially to part 2, where concussions as well as intracranial hemorrhaging were most rare (*P* = 0.033). 

With 64%, rib fractures were the most common injury associated with a sternum fracture (*n* = 37). Of these, serial rib fractures were found in 24 cases (=65%), and the rest were solitary rib fractures. In almost half of the cases where a rib fracture or serial rib fracture was found, the concomitant sternum fracture was located at the manubrium (*n* = 16, 43%). Clavicle fractures were found in six cases (10%). In eight cases, a fracture of the scapula was found, whereby most of these could be observed in cases of manubrium sterni fracture (*P* = 0.021). Pneumothoraces occurred in 7 cases and lung tissue injuries were found in 20 cases. No significant differences could be observed within the different topographic zones. 

Five patients suffered from a cardiac contusion. Of these, four out of five were injured in a car accident. Three of these patients presented with dysrhythmia and in three cases a heart specific enzyme increase was found. The troponin T ranged between 160 ug/L and 537 ug/L and CKMB between 0.072 ug/L and 0.17 ug/L.

With 57%, a spinal fracture represented the second most frequent concomitant injury. A total of 51 spine fractures in 33 patients were detected. Five patients suffered from a fracture of both the thoracic and lumbar spine. There were four cases of a combined cervical and thoracic spine fracture, two cases of a cervical and lumbar fracture, and finally four patients with a fracture of the cervical, thoracic, and also lumbar spine. All four patients were admitted to the ICU and only one survived. Three out of four of the patients had sustained a fracture of the manubrium sterni. 

In patients with a concomitant spinal fracture, the topographic analysis of the sternal fracture indicated a statistically significant higher rate of thoracic spine lesions in cases of fractures of the manubrium sterni (61.9%; *P* = 0.005) (Table 3). The severity of such a thoracic spine fracture was different depending on the sternum fracture location: whereas in fractures of the manubrium sterni five of the thirteen thoracic spine fractures could be classified as flexion distraction fractures (AO type B), only one was observed in patients with a fracture of part 3 of the corpus sterni. Fractures of the manubrium sterni were furthermore strongly associated with other injuries to the chest and head (Table 3).

Lesions of intra-abdominal organs were found in combination with all sternal zones, whereby higher rates could be observed in cases of corpus sterni fracture part 3 and fractures of the manubrium sterni (Table 3). In this context no significant differences could be observed within the different topographic zones. Involved abdominal organs were in descending order: the spleen (*n* = 6), liver (*n* = 2) and kidneys (*n* = 2) as well as the adrenal glands (*n* = 1), ovarian (*n* = 1), stomach (*n* = 1), and the abdominal aorta (*n* = 1).

## 4. Discussion

The incidence of sternal fractures after trauma appears to be rare; nevertheless it has increased over the past several decades [1, 2, 20–22]. For instance, an analysis of 1,124 motor vehicle collision victims in a three-year period showed an increase of sternal fractures from 0.7% to 4% [21]. In this analysis, as well as in others, the increase has mainly been associated with the introduction of seat belts [21, 23–25]. This observation leads to the expression seat belt syndrome for sternal fractures [26]. Nearly all studies involving more than fifty sternal fractures assume that this is due mainly to frontal collisions as a primary cause [17, 25]. In our study, over 40% of the sternal fractures occurred in victims of motor vehicle crashes. However, we have no information how many of these were wearing seat belts. 

**Figure 2:**
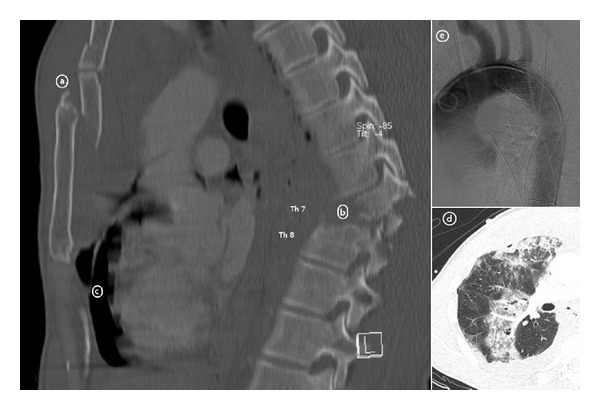
Multiple injured 38-year old patient after crash with paraglider. In addition to the sternum fracture of the manubrium and part 1 of the corpus (a), he suffered from a type b fracture of the thoracic spine with paraplegia (b), a chest trauma with rip fractures, and injuries to the lung parenchyma and lung contusions (d) as well as a thoracic aortic rupture (e).

Falls from a height were the second most common injury mechanism leading to sternal fractures (Figure 2). Past studies have shown that the mean height of such a fall was greater than five meters. The sternal fracture occurs as a consequence of a considerable direct external force or as a result of a vertebral compression and flexion of the chest [27]. All other observed mechanisms of injury could be attributed to direct external force.

A possible reason for the high mortality rate (15.5%) in our survey was due to the large proportion of severe chest and brain injuries. This is underpinned by the mean ISS of 20.5 representing the high rate of severely injured patients, which in turn leads to a high rate of intensive care unit admissions (56.8%). In the literature, the primary mortality from blunt chest trauma lies between 15 and 25% and can significantly increase the overall mortality in multiple injured patients [28, 29]. This overall mortality found in our investigation appears to be in line with this observation. Lower mortality rates in recent other studies seem to reflect a different mechanism and pattern of injury as well as different population groups being involved [8, 30, 31]. 

When comparing the survival rate to the level of a sternal fracture, it is remarkable that the highest mortality rate was found in patients with a fracture of the manubrium sterni (23.8%, *n* = 5). 

Although the majority of sternal fractures can be treated with conservative methods [2, 21, 32], their identification should raise suspicion for other associated injuries. In the present cohort, only five patients were admitted with isolated sternal fractures; all others suffered from additional injuries. Compared to previous results by other investigators, we found the most common concomitant injuries in patients with sternal fractures to be rib fractures [1–3]. In a cadaver study, it was recently found that the rib cage and sternum provide 40% of the stability to the thoracic spine in flexion extension, 22% in lateral bending, and 15% in axial rotation [33]. Therefore, the combination of a fracture of the sternum and a rib fracture decreases the stability of the thorax dramatically, especially in the presence of serial rib fractures. In this context, Berg postulated that the sternum rib complex stabilizes the thoracic spine as a forth column [7]. The high incidence of serial rib fractures in our patient cohort with a consecutive decrease of stability might explain the high incidence of thoracic spine injuries. Numerous previous investigations demonstrated a strong correlation between sternal fractures and a thoracic spine injury. In our study, patients with a fracture of the manubrium sterni suffered from concomitant injuries of the thoracic spine in 61% of the cases. The incidence of thoracic spine fractures as a concomitant injury when another (lower) level of the sternum was fractured decreased steadily from 36% in part 1 to 9% in part 3 sternum fractures (Table 1). In the literature, such fractures were found to be due to a postulated hyperflexion mechanism as the predominant cause of injury [7, 14]. This is in concordance with our present cohort, where nearly 50% of all thoracic spine injuries could be classified as hyperflexion fractures (AO type B). Half of these type B fractures were associated with a fracture of the manubrium sterni. 

Although, the division of sternum in four topographic zones is artificial and normally will not be practiced in clinical settings, this result supports previous studies which call for extensive diagnostic efforts to rule out occult fractures of the spine when a fracture of the manubrium sterni, is present [30].

Similarly, a high incidence of accompanying cervical spine injuries in cases of manubrium sterni fractures could be observed. Nearly one-third of all patients with a fracture of the manubrium sterni presented a lesion of the cervical spine. With a fracture of any part of the corpus sterni the incidence was much lower. Of all cervical spine injuries in the present cohort (*n* = 11), 54% (*n* = 6) were associated with a fracture of the manubrium sterni. Therefore, the available data demonstrates a clear correlation between cervical spine injuries and the level of a sternal fracture.

Contrary to the low association of part 3 sternum fractures with lesions of the cervical and thoracic spine, the incidence of lumbar spine injuries was comparatively high (54%). 

These findings suggest that in sternum injuries, beside the traditionally accepted belief that the upper thoracic spine is primarily affected, lumbar and cervical spine injuries may be associated as well.

The close proximity of the sternum to underlying organs of the thorax requires the evaluation of these structures to rule out further injuries. Besides the aforementioned, a pulmonary contusion was the third most common observed injury of the chest, an injury that has been reported as having a mortality of as high as 35% in the multiple injured patient [34]. There was no significant difference in the rate of occurrence of a pulmonary contusion depending on sternum fracture topography. A total of five (9%) of our patients showed cardiac abnormalities; three patients suffered from posttraumatic arrhythmia and three presented elevated heart enzymes (troponin, CKMB). In one case a pericardial effusion was found. The incidence is in line with previous investigations on blunt trauma to the chest [3, 25]. However, the significance of a cardiac affection in blunt chest trauma is controversially discussed in the literature. Whereas in former investigations sternal fractures were frequently considered as an indicator of possible injuries of the heart [13, 18, 35], this view has increasingly been questioned in the recent past [36–38]. Besides the observed arrhythmia, elevated heart enzymes and pericardial effusion, a cardiac malfunction leading to further clinical consequences was not observed in any of our cases. Therefore, we also tend to the opinion that a sternal fracture is not a relevant marker for cardiac lesions in blunt thoracic trauma [39]. Concerning the sternal fracture level, an equal distribution between fractures of the manubrium sterni, part 3 and part 2 of the corpus sterni could be recorded. A thoracic aortic injury was also noted in two cases. This is a relatively low prevalence compared to previous investigations [3, 39]. However, no investigations exist about the incidence of preclinical death of patients with a sternal fracture and accompanying thoracic aortic injuries.

In the examined population of patients with sternal fractures, the most common concomitant extrathoracic injuries were the involvement of the brain in 48.3% of the cases (*n* = 28). More than half of these patients (*n* = 15) presented typical signs of a concussion; in all other cases (*n* = 12) an intracerebral hemorrhage could be detected. The latter was most commonly observed among victims of car and motorcycle accidents (*n* = 8). It should be noted that the incidence of accompanying brain injuries was significantly highest in the cohort which sustained a fracture of the manubrium sterni (67%; *n* = 14). This cohort also showed the highest proportion of severe brain injuries (29%; *n* = 6).

We acknowledge several limitations of the present study. First, due to the retrospective study design we depended on complete and accurate patient medical charts to evaluate the physical condition on admission. However, data collection was done in a routine setting by trained personal of the trauma center and we could not ensure with final certainty the completeness of data. With regard to concomitant injuries CT scans were assessed again without knowledge of previous findings. Therefore completeness could be ensured. Second, the study was undertaken at a single designated trauma centre. This might have introduced selection bias and limited the external validity of the findings. Third, the low numbers of fractures make interpretation difficult. Therefore, no regression modelling was possible to evaluate interactions between the injuries. 

In conclusion and besides these limitations our study demonstrated that sternal fractures are rare but serious injuries of the chest wall due to high rate of concomitant injuries, including severe thoracic spine as well as brain injuries. Therefore, whole body CT scans should be performed in all cases with adequate trauma and suspicion of sternal fracture to detect the frequently associated injuries and reduce the increased mortality. Further, fracture location can provide certain important information regarding concomitant injuries. This is illustrated by the fact that fractures of the manubrium sterni had the highest rate of concomitant injuries compared to the other locations. 

## Figures and Tables

**Table 1:** Mechanisms of injury.

Motor vehicle collisions	*n* = 25(43.1%)
Fall from a height	*n* = 19 (32.8%)
<2 metre	*n* = 4
2–5 metre	*n* = 5
5–10 metre	*n* = 6
>10 metre	*n* = 4
Motorcycle accidents	*n* = 8 (13.8%)
Pedestrians	*n* = 4 (6.9%)
Violent assaults	*n* = 2 (3.45%)

**Table 2:** Associated thoracic and extrathoracic injuries.

Associated thoracic injuries		Associated extrathoracic injuries	
Rib fractures	*n* = 37 (63.8%)	Traumatic brain injury	*n* = 28 (48.3%)
Isolatet rib fracture	*n* = 13 (34%)	Concussion	*n* = 15 (53.6%)
Serial rib fracture	*n* = 24 (65%)	Intracranial bleeding	*n* = 13 (46.4%)
Thoracic spine injury	*n* = 22 (38%)	Lumbar spine injury	*n* = 16 (27.6%)
Lung contusion	*n* = 16 (22.4%)	Abdominal injury	*n* = 16 (11%)
Scapula fracture	*n* = 8 (13.8%)	Cervical spine injury	*n* = 13 (22.4%)
Cardiac contusion	*n* = 5 (8.6%)	Pelvic fracture	*n* = 9 (15.5%)
Pneumothorax	*n* = 7 (12.1%)		
Clavicula fracture	*n* = 6 (10.3%)		
Thoracic aortic rupture	*n* = 2 (3.4%)		

**Table 3:** Associated injuries with regard to fracture location.

	*n*	Mean ISS	CSL	TSL	LSL	RF	LPI	LC	SF	CF	TBI	AL
Manubrium sterni	21	23	6 (29%)	**13 **(62%)*	6 (29%)	16 (76%)	9 (43%)	8 (38%)	**6 **(29%)*	4 (19%)	**14 **(67%)*	4 (19%)
Corpus sterni part 1	11	16	4 (36%)	4 (37%)	1 (9%)	6 (55%)	3 (27%)	2 (18%)	0	2 (18%)	5 (46%)	2 (18%)
Corpus sterni part 2	15	22	1 (7%)	4 (27%)	3 (20%)	9 (60%)	5 (33%)	4 (27%)	1 (7%)	0	4 (27%)	2 (13%)
Corpus sterni part 3	11	20	2 (18%)	1 (9%)	**6 **(55%)*	6 (55%)	3 (27%)	2 (18%)	1 (9%)	0	5 (46%)	3 (27%)
*P* values			*P* > 0.05	*P* = 0.005	*P* = 0.041	*P* > 0.05	*P* > 0.05	*P* > 0.05	*P* = 0.021	*P* > 0.05	*P* = 0.033	*P* > 0.05

CSL: cervical spine lesion; TSL: thoracic spine lesion; LSL: lumbar spine lesion; RF: rib fracture; LPI: lung parenchymal injury; LC: lung contusion; SF: scapula fracture; CF: clavicle fracture; TBI: traumatic brain injury; AL: abdominal lesion (*statistical significance).

**Table 4:** Intensive care unit (ICU) admission and mortality with regard to fracture location.

	ICU	Mortality
Manubrium sterni	**16 **(76.2%)*	5 (23.8%)
Corpus sterni part 1	5 (45.5%)	0
Corpus sterni part 2	6 (40%)	3 (20%)
Corpus sterni part 3	6 (54.4%)	1 (9.1%)
*P* value	*P* = 0.024	*P* > 0.05

*Statistical significance.
